# Supplementary material for: Microsporidia infection alters C. elegans lipid levels
Source: PLoS One. 2025 Jul 1;20(7):e0327188. doi: 10.1371/journal.pone.0327188 (PMC12212544; doi:10.1371/journal.pone.0327188)
Supplement: S1 Fig — (PDF) [file pone.0327188.s001.pdf]

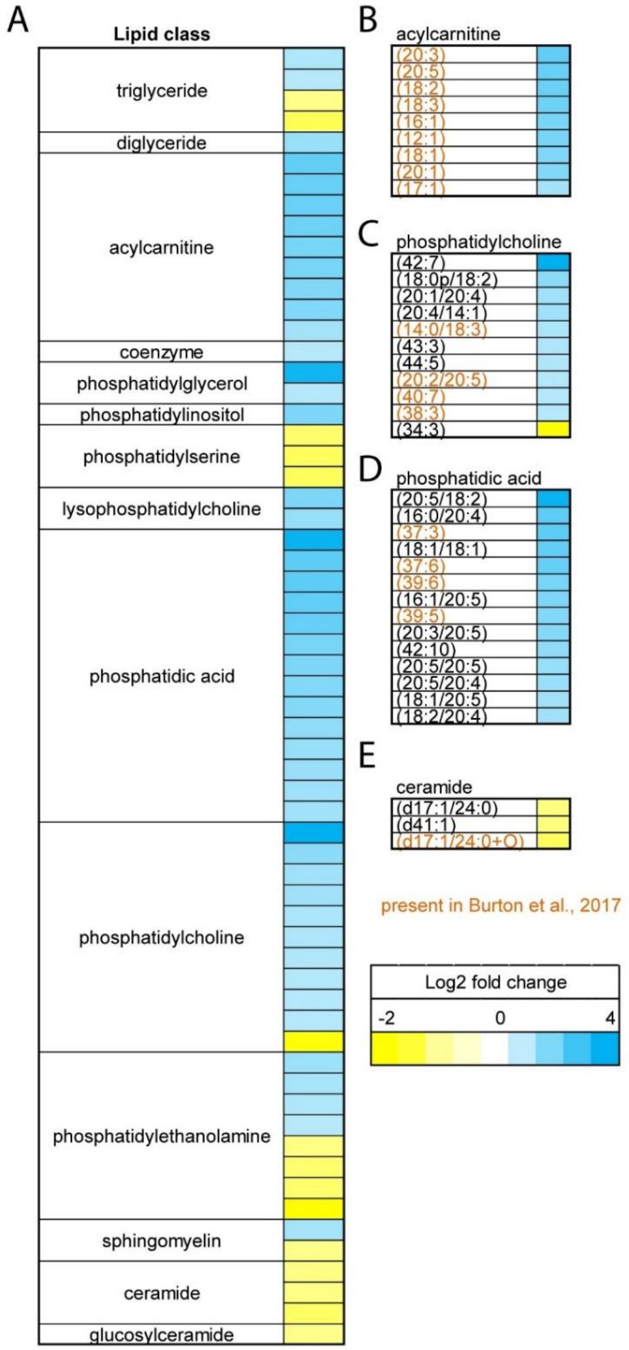

S1 Fig. *N. parisii* infection of *C. elegans* parents alters lipids in offspring.

(A-E) *C. elegans* were infected for 72 hours and were harvested using sodium hypochlorite treatment. After 3 hours embryos were harvested and lipids measured using LC/MS. Heat map showing lipid metabolites that are upregulated or downregulated in embryos from infected parents by more than 2-fold with a significant p-value ($p < 0.01$). (A) All classes of lipids significantly upregulated or downregulated. (B) Acylcarnitines. (C) Phosphatidylcholines. (D) Phosphatidic acids. (E) Ceramides. (B-E) Lipid species detected in Burton et al. 2017 colored in orange [55].
