# Supplementary material for: Microsporidia infection alters C. elegans lipid levels
Source: PLoS One. 2025 Jul 1;20(7):e0327188. doi: 10.1371/journal.pone.0327188 (PMC12212544; doi:10.1371/journal.pone.0327188)
Supplement: S2 Table — (PDF) [file pone.0327188.s003.pdf]

Table S2. List of *C. elegans* strains used in the study.

Strain	Genotype
**N2**	N2
**VS20**	*hjIs67 [atgl-1p::atgl-1::GFP + mec-7::RFP]*
**VC20458**	*atgl-1 (gk176565) [P87S] III*
**RG3279**	*asah-1 (ve779 [LoxP + myo-2p::GFP::unc-54 3' UTR + rps-27p::neoR::unc-54 3' UTR + LoxP]) I*
RB782	*asah-2 (ok564) II*
RB1465	*sptl-1 (ok1693) II*
RB1579	*sptl-3 (ok1927) V*
RB1036	*hyl-1 (ok976) IV*
VC334	*hyl-1 (gk203) IV*
RB1498	*hyl-2 (ok1766) X*
RB1685	*ttm-5 (ok2095) I*
RB2135	*F33D4.4 (ok2843) IV*
VC507	*lagr-1 (gk263) I*
VC765	*lagr-1 (gk331) I*
